# Ensembl 2024

**DOI:** 10.1093/nar/gkad1049

**Published:** 2023-11-11

**Authors:** Peter W Harrison, M Ridwan Amode, Olanrewaju Austine-Orimoloye, Andrey G Azov, Matthieu Barba, If Barnes, Arne Becker, Ruth Bennett, Andrew Berry, Jyothish Bhai, Simarpreet Kaur Bhurji, Sanjay Boddu, Paulo R Branco Lins, Lucy Brooks, Shashank Budhanuru Ramaraju, Lahcen I Campbell, Manuel Carbajo Martinez, Mehrnaz Charkhchi, Kapeel Chougule, Alexander Cockburn, Claire Davidson, Nishadi H De Silva, Kamalkumar Dodiya, Sarah Donaldson, Bilal El Houdaigui, Tamara El Naboulsi, Reham Fatima, Carlos Garcia Giron, Thiago Genez, Dionysios Grigoriadis, Gurpreet S Ghattaoraya, Jose Gonzalez Martinez, Tatiana A Gurbich, Matthew Hardy, Zoe Hollis, Thibaut Hourlier, Toby Hunt, Mike Kay, Vinay Kaykala, Tuan Le, Diana Lemos, Disha Lodha, Diego Marques-Coelho, Gareth Maslen, Gabriela Alejandra Merino, Louisse Paola Mirabueno, Aleena Mushtaq, Syed Nakib Hossain, Denye N Ogeh, Manoj Pandian Sakthivel, Anne Parker, Malcolm Perry, Ivana Piližota, Daniel Poppleton, Irina Prosovetskaia, Shriya Raj, José G Pérez-Silva, Ahamed Imran Abdul Salam, Shradha Saraf, Nuno Saraiva-Agostinho, Dan Sheppard, Swati Sinha, Botond Sipos, Vasily Sitnik, William Stark, Emily Steed, Marie-Marthe Suner, Likhitha Surapaneni, Kyösti Sutinen, Francesca Floriana Tricomi, David Urbina-Gómez, Andres Veidenberg, Thomas A Walsh, Doreen Ware, Elizabeth Wass, Natalie L Willhoft, Jamie Allen, Jorge Alvarez-Jarreta, Marc Chakiachvili, Bethany Flint, Stefano Giorgetti, Leanne Haggerty, Garth R Ilsley, Jon Keatley, Jane E Loveland, Benjamin Moore, Jonathan M Mudge, Guy Naamati, John Tate, Stephen J Trevanion, Andrea Winterbottom, Adam Frankish, Sarah E Hunt, Fiona Cunningham, Sarah Dyer, Robert D Finn, Fergal J Martin, Andrew D Yates

**Affiliations:** European Molecular Biology Laboratory, European Bioinformatics Institute, Wellcome Genome Campus, Hinxton, Cambridge, Cambridgeshire CB10 1SD, UK; European Molecular Biology Laboratory, European Bioinformatics Institute, Wellcome Genome Campus, Hinxton, Cambridge, Cambridgeshire CB10 1SD, UK; European Molecular Biology Laboratory, European Bioinformatics Institute, Wellcome Genome Campus, Hinxton, Cambridge, Cambridgeshire CB10 1SD, UK; European Molecular Biology Laboratory, European Bioinformatics Institute, Wellcome Genome Campus, Hinxton, Cambridge, Cambridgeshire CB10 1SD, UK; European Molecular Biology Laboratory, European Bioinformatics Institute, Wellcome Genome Campus, Hinxton, Cambridge, Cambridgeshire CB10 1SD, UK; European Molecular Biology Laboratory, European Bioinformatics Institute, Wellcome Genome Campus, Hinxton, Cambridge, Cambridgeshire CB10 1SD, UK; European Molecular Biology Laboratory, European Bioinformatics Institute, Wellcome Genome Campus, Hinxton, Cambridge, Cambridgeshire CB10 1SD, UK; European Molecular Biology Laboratory, European Bioinformatics Institute, Wellcome Genome Campus, Hinxton, Cambridge, Cambridgeshire CB10 1SD, UK; European Molecular Biology Laboratory, European Bioinformatics Institute, Wellcome Genome Campus, Hinxton, Cambridge, Cambridgeshire CB10 1SD, UK; European Molecular Biology Laboratory, European Bioinformatics Institute, Wellcome Genome Campus, Hinxton, Cambridge, Cambridgeshire CB10 1SD, UK; European Molecular Biology Laboratory, European Bioinformatics Institute, Wellcome Genome Campus, Hinxton, Cambridge, Cambridgeshire CB10 1SD, UK; European Molecular Biology Laboratory, European Bioinformatics Institute, Wellcome Genome Campus, Hinxton, Cambridge, Cambridgeshire CB10 1SD, UK; European Molecular Biology Laboratory, European Bioinformatics Institute, Wellcome Genome Campus, Hinxton, Cambridge, Cambridgeshire CB10 1SD, UK; European Molecular Biology Laboratory, European Bioinformatics Institute, Wellcome Genome Campus, Hinxton, Cambridge, Cambridgeshire CB10 1SD, UK; European Molecular Biology Laboratory, European Bioinformatics Institute, Wellcome Genome Campus, Hinxton, Cambridge, Cambridgeshire CB10 1SD, UK; European Molecular Biology Laboratory, European Bioinformatics Institute, Wellcome Genome Campus, Hinxton, Cambridge, Cambridgeshire CB10 1SD, UK; European Molecular Biology Laboratory, European Bioinformatics Institute, Wellcome Genome Campus, Hinxton, Cambridge, Cambridgeshire CB10 1SD, UK; European Molecular Biology Laboratory, European Bioinformatics Institute, Wellcome Genome Campus, Hinxton, Cambridge, Cambridgeshire CB10 1SD, UK; Cold Spring Harbor Laboratory, 1 Bungtown Rd, Cold Spring Harbor, NY 11724, USA; European Molecular Biology Laboratory, European Bioinformatics Institute, Wellcome Genome Campus, Hinxton, Cambridge, Cambridgeshire CB10 1SD, UK; European Molecular Biology Laboratory, European Bioinformatics Institute, Wellcome Genome Campus, Hinxton, Cambridge, Cambridgeshire CB10 1SD, UK; European Molecular Biology Laboratory, European Bioinformatics Institute, Wellcome Genome Campus, Hinxton, Cambridge, Cambridgeshire CB10 1SD, UK; European Molecular Biology Laboratory, European Bioinformatics Institute, Wellcome Genome Campus, Hinxton, Cambridge, Cambridgeshire CB10 1SD, UK; European Molecular Biology Laboratory, European Bioinformatics Institute, Wellcome Genome Campus, Hinxton, Cambridge, Cambridgeshire CB10 1SD, UK; European Molecular Biology Laboratory, European Bioinformatics Institute, Wellcome Genome Campus, Hinxton, Cambridge, Cambridgeshire CB10 1SD, UK; European Molecular Biology Laboratory, European Bioinformatics Institute, Wellcome Genome Campus, Hinxton, Cambridge, Cambridgeshire CB10 1SD, UK; European Molecular Biology Laboratory, European Bioinformatics Institute, Wellcome Genome Campus, Hinxton, Cambridge, Cambridgeshire CB10 1SD, UK; European Molecular Biology Laboratory, European Bioinformatics Institute, Wellcome Genome Campus, Hinxton, Cambridge, Cambridgeshire CB10 1SD, UK; European Molecular Biology Laboratory, European Bioinformatics Institute, Wellcome Genome Campus, Hinxton, Cambridge, Cambridgeshire CB10 1SD, UK; European Molecular Biology Laboratory, European Bioinformatics Institute, Wellcome Genome Campus, Hinxton, Cambridge, Cambridgeshire CB10 1SD, UK; European Molecular Biology Laboratory, European Bioinformatics Institute, Wellcome Genome Campus, Hinxton, Cambridge, Cambridgeshire CB10 1SD, UK; European Molecular Biology Laboratory, European Bioinformatics Institute, Wellcome Genome Campus, Hinxton, Cambridge, Cambridgeshire CB10 1SD, UK; European Molecular Biology Laboratory, European Bioinformatics Institute, Wellcome Genome Campus, Hinxton, Cambridge, Cambridgeshire CB10 1SD, UK; European Molecular Biology Laboratory, European Bioinformatics Institute, Wellcome Genome Campus, Hinxton, Cambridge, Cambridgeshire CB10 1SD, UK; European Molecular Biology Laboratory, European Bioinformatics Institute, Wellcome Genome Campus, Hinxton, Cambridge, Cambridgeshire CB10 1SD, UK; European Molecular Biology Laboratory, European Bioinformatics Institute, Wellcome Genome Campus, Hinxton, Cambridge, Cambridgeshire CB10 1SD, UK; European Molecular Biology Laboratory, European Bioinformatics Institute, Wellcome Genome Campus, Hinxton, Cambridge, Cambridgeshire CB10 1SD, UK; European Molecular Biology Laboratory, European Bioinformatics Institute, Wellcome Genome Campus, Hinxton, Cambridge, Cambridgeshire CB10 1SD, UK; European Molecular Biology Laboratory, European Bioinformatics Institute, Wellcome Genome Campus, Hinxton, Cambridge, Cambridgeshire CB10 1SD, UK; European Molecular Biology Laboratory, European Bioinformatics Institute, Wellcome Genome Campus, Hinxton, Cambridge, Cambridgeshire CB10 1SD, UK; European Molecular Biology Laboratory, European Bioinformatics Institute, Wellcome Genome Campus, Hinxton, Cambridge, Cambridgeshire CB10 1SD, UK; European Molecular Biology Laboratory, European Bioinformatics Institute, Wellcome Genome Campus, Hinxton, Cambridge, Cambridgeshire CB10 1SD, UK; European Molecular Biology Laboratory, European Bioinformatics Institute, Wellcome Genome Campus, Hinxton, Cambridge, Cambridgeshire CB10 1SD, UK; European Molecular Biology Laboratory, European Bioinformatics Institute, Wellcome Genome Campus, Hinxton, Cambridge, Cambridgeshire CB10 1SD, UK; European Molecular Biology Laboratory, European Bioinformatics Institute, Wellcome Genome Campus, Hinxton, Cambridge, Cambridgeshire CB10 1SD, UK; European Molecular Biology Laboratory, European Bioinformatics Institute, Wellcome Genome Campus, Hinxton, Cambridge, Cambridgeshire CB10 1SD, UK; European Molecular Biology Laboratory, European Bioinformatics Institute, Wellcome Genome Campus, Hinxton, Cambridge, Cambridgeshire CB10 1SD, UK; European Molecular Biology Laboratory, European Bioinformatics Institute, Wellcome Genome Campus, Hinxton, Cambridge, Cambridgeshire CB10 1SD, UK; European Molecular Biology Laboratory, European Bioinformatics Institute, Wellcome Genome Campus, Hinxton, Cambridge, Cambridgeshire CB10 1SD, UK; European Molecular Biology Laboratory, European Bioinformatics Institute, Wellcome Genome Campus, Hinxton, Cambridge, Cambridgeshire CB10 1SD, UK; European Molecular Biology Laboratory, European Bioinformatics Institute, Wellcome Genome Campus, Hinxton, Cambridge, Cambridgeshire CB10 1SD, UK; European Molecular Biology Laboratory, European Bioinformatics Institute, Wellcome Genome Campus, Hinxton, Cambridge, Cambridgeshire CB10 1SD, UK; European Molecular Biology Laboratory, European Bioinformatics Institute, Wellcome Genome Campus, Hinxton, Cambridge, Cambridgeshire CB10 1SD, UK; European Molecular Biology Laboratory, European Bioinformatics Institute, Wellcome Genome Campus, Hinxton, Cambridge, Cambridgeshire CB10 1SD, UK; European Molecular Biology Laboratory, European Bioinformatics Institute, Wellcome Genome Campus, Hinxton, Cambridge, Cambridgeshire CB10 1SD, UK; European Molecular Biology Laboratory, European Bioinformatics Institute, Wellcome Genome Campus, Hinxton, Cambridge, Cambridgeshire CB10 1SD, UK; European Molecular Biology Laboratory, European Bioinformatics Institute, Wellcome Genome Campus, Hinxton, Cambridge, Cambridgeshire CB10 1SD, UK; European Molecular Biology Laboratory, European Bioinformatics Institute, Wellcome Genome Campus, Hinxton, Cambridge, Cambridgeshire CB10 1SD, UK; European Molecular Biology Laboratory, European Bioinformatics Institute, Wellcome Genome Campus, Hinxton, Cambridge, Cambridgeshire CB10 1SD, UK; European Molecular Biology Laboratory, European Bioinformatics Institute, Wellcome Genome Campus, Hinxton, Cambridge, Cambridgeshire CB10 1SD, UK; European Molecular Biology Laboratory, European Bioinformatics Institute, Wellcome Genome Campus, Hinxton, Cambridge, Cambridgeshire CB10 1SD, UK; European Molecular Biology Laboratory, European Bioinformatics Institute, Wellcome Genome Campus, Hinxton, Cambridge, Cambridgeshire CB10 1SD, UK; European Molecular Biology Laboratory, European Bioinformatics Institute, Wellcome Genome Campus, Hinxton, Cambridge, Cambridgeshire CB10 1SD, UK; European Molecular Biology Laboratory, European Bioinformatics Institute, Wellcome Genome Campus, Hinxton, Cambridge, Cambridgeshire CB10 1SD, UK; European Molecular Biology Laboratory, European Bioinformatics Institute, Wellcome Genome Campus, Hinxton, Cambridge, Cambridgeshire CB10 1SD, UK; European Molecular Biology Laboratory, European Bioinformatics Institute, Wellcome Genome Campus, Hinxton, Cambridge, Cambridgeshire CB10 1SD, UK; European Molecular Biology Laboratory, European Bioinformatics Institute, Wellcome Genome Campus, Hinxton, Cambridge, Cambridgeshire CB10 1SD, UK; European Molecular Biology Laboratory, European Bioinformatics Institute, Wellcome Genome Campus, Hinxton, Cambridge, Cambridgeshire CB10 1SD, UK; European Molecular Biology Laboratory, European Bioinformatics Institute, Wellcome Genome Campus, Hinxton, Cambridge, Cambridgeshire CB10 1SD, UK; European Molecular Biology Laboratory, European Bioinformatics Institute, Wellcome Genome Campus, Hinxton, Cambridge, Cambridgeshire CB10 1SD, UK; European Molecular Biology Laboratory, European Bioinformatics Institute, Wellcome Genome Campus, Hinxton, Cambridge, Cambridgeshire CB10 1SD, UK; European Molecular Biology Laboratory, European Bioinformatics Institute, Wellcome Genome Campus, Hinxton, Cambridge, Cambridgeshire CB10 1SD, UK; European Molecular Biology Laboratory, European Bioinformatics Institute, Wellcome Genome Campus, Hinxton, Cambridge, Cambridgeshire CB10 1SD, UK; Cold Spring Harbor Laboratory, 1 Bungtown Rd, Cold Spring Harbor, NY 11724, USA; USDA ARS NAA Robert W. Holley Center for Agriculture and Health, Agricultural Research Service, Ithaca, NY 14853, USA; European Molecular Biology Laboratory, European Bioinformatics Institute, Wellcome Genome Campus, Hinxton, Cambridge, Cambridgeshire CB10 1SD, UK; European Molecular Biology Laboratory, European Bioinformatics Institute, Wellcome Genome Campus, Hinxton, Cambridge, Cambridgeshire CB10 1SD, UK; European Molecular Biology Laboratory, European Bioinformatics Institute, Wellcome Genome Campus, Hinxton, Cambridge, Cambridgeshire CB10 1SD, UK; European Molecular Biology Laboratory, European Bioinformatics Institute, Wellcome Genome Campus, Hinxton, Cambridge, Cambridgeshire CB10 1SD, UK; European Molecular Biology Laboratory, European Bioinformatics Institute, Wellcome Genome Campus, Hinxton, Cambridge, Cambridgeshire CB10 1SD, UK; European Molecular Biology Laboratory, European Bioinformatics Institute, Wellcome Genome Campus, Hinxton, Cambridge, Cambridgeshire CB10 1SD, UK; European Molecular Biology Laboratory, European Bioinformatics Institute, Wellcome Genome Campus, Hinxton, Cambridge, Cambridgeshire CB10 1SD, UK; European Molecular Biology Laboratory, European Bioinformatics Institute, Wellcome Genome Campus, Hinxton, Cambridge, Cambridgeshire CB10 1SD, UK; European Molecular Biology Laboratory, European Bioinformatics Institute, Wellcome Genome Campus, Hinxton, Cambridge, Cambridgeshire CB10 1SD, UK; European Molecular Biology Laboratory, European Bioinformatics Institute, Wellcome Genome Campus, Hinxton, Cambridge, Cambridgeshire CB10 1SD, UK; European Molecular Biology Laboratory, European Bioinformatics Institute, Wellcome Genome Campus, Hinxton, Cambridge, Cambridgeshire CB10 1SD, UK; European Molecular Biology Laboratory, European Bioinformatics Institute, Wellcome Genome Campus, Hinxton, Cambridge, Cambridgeshire CB10 1SD, UK; European Molecular Biology Laboratory, European Bioinformatics Institute, Wellcome Genome Campus, Hinxton, Cambridge, Cambridgeshire CB10 1SD, UK; European Molecular Biology Laboratory, European Bioinformatics Institute, Wellcome Genome Campus, Hinxton, Cambridge, Cambridgeshire CB10 1SD, UK; European Molecular Biology Laboratory, European Bioinformatics Institute, Wellcome Genome Campus, Hinxton, Cambridge, Cambridgeshire CB10 1SD, UK; European Molecular Biology Laboratory, European Bioinformatics Institute, Wellcome Genome Campus, Hinxton, Cambridge, Cambridgeshire CB10 1SD, UK; European Molecular Biology Laboratory, European Bioinformatics Institute, Wellcome Genome Campus, Hinxton, Cambridge, Cambridgeshire CB10 1SD, UK; European Molecular Biology Laboratory, European Bioinformatics Institute, Wellcome Genome Campus, Hinxton, Cambridge, Cambridgeshire CB10 1SD, UK; European Molecular Biology Laboratory, European Bioinformatics Institute, Wellcome Genome Campus, Hinxton, Cambridge, Cambridgeshire CB10 1SD, UK; European Molecular Biology Laboratory, European Bioinformatics Institute, Wellcome Genome Campus, Hinxton, Cambridge, Cambridgeshire CB10 1SD, UK; European Molecular Biology Laboratory, European Bioinformatics Institute, Wellcome Genome Campus, Hinxton, Cambridge, Cambridgeshire CB10 1SD, UK; European Molecular Biology Laboratory, European Bioinformatics Institute, Wellcome Genome Campus, Hinxton, Cambridge, Cambridgeshire CB10 1SD, UK; European Molecular Biology Laboratory, European Bioinformatics Institute, Wellcome Genome Campus, Hinxton, Cambridge, Cambridgeshire CB10 1SD, UK; European Molecular Biology Laboratory, European Bioinformatics Institute, Wellcome Genome Campus, Hinxton, Cambridge, Cambridgeshire CB10 1SD, UK

## Abstract

Ensembl (https://www.ensembl.org) is a freely available genomic resource that has produced high-quality annotations, tools, and services for vertebrates and model organisms for more than two decades. In recent years, there has been a dramatic shift in the genomic landscape, with a large increase in the number and phylogenetic breadth of high-quality reference genomes, alongside major advances in the pan-genome representations of higher species. In order to support these efforts and accelerate downstream research, Ensembl continues to focus on scaling for the rapid annotation of new genome assemblies, developing new methods for comparative analysis, and expanding the depth and quality of our genome annotations. This year we have continued our expansion to support global biodiversity research, doubling the number of annotated genomes we support on our Rapid Release site to over 1700, driven by our close collaboration with biodiversity projects such as Darwin Tree of Life. We have also strengthened support for key agricultural species, including the first regulatory builds for farmed animals, and have updated key tools and resources that support the global scientific community, notably the Ensembl Variant Effect Predictor. Ensembl data, software, and tools are freely available.

## Graphical Abstract

**Graphical Abstract:**
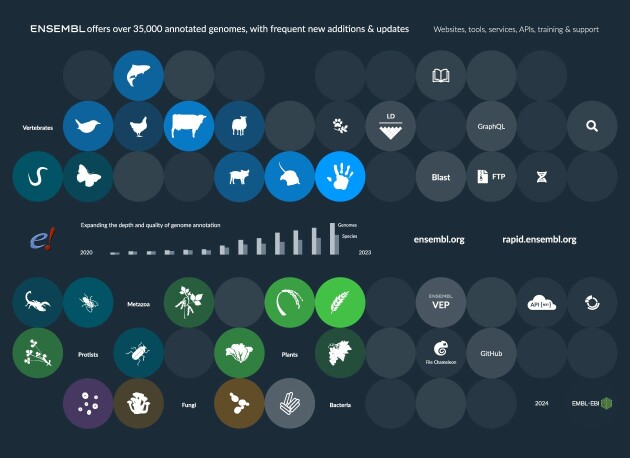

## Introduction

Ensembl (https://www.ensembl.org) is a freely available platform for exploring sequences and genome annotations across the tree of life, producing high-quality genomic resources and tools for vertebrate and non-vertebrate species. For >20 years, Ensembl has developed infrastructure to deliver reference genome assemblies from public archives for the genomic interpretation of genes, regulatory regions, variants, and comparative data. Ensembl is recognised as a Global Core Biodata Resource and ELIXIR Core Data Resource (1) highlighting the fundamental role it plays in life sciences research for long-term preservation and open access to high-quality annotated genomic data. Annotation datasets are available interactively through the Ensembl genome browser website, programmatically through our Application Programming Interfaces (API) and independently as downloadable files. In recent years, we have supported a parallel mechanism for a more rapid release of reference genomes. This Rapid Release site (https://rapid.ensembl.org/) (2,3) is updated every two weeks, and now, with >50 releases, it has been crucial for the timely release of annotations for biodiversity projects such as Darwin Tree of Life (4). The site supports >1700 annotations from >1000 different species, a twofold increase in the last year. While we have significantly expanded our annotations for species across the tree of life, we continue to provide a rich collection of resources for key species and taxonomic groups. This includes important new annotations to support food security research with the first Ensembl regulatory builds, beyond human and mouse, in farmed animals.

We have continued to update our suite of Ensembl tools, most notably with improvements to the Ensembl Variant Effect Predictor (5) (VEP), which calculates the expected effect of user-supplied variants on genes, transcripts, regulatory regions, and protein sequence. We also provide an update on the development of our new re-envisioned Ensembl platform, currently in beta release (https://beta.ensembl.org/), for the future management, browsing, analysis and dissemination of genomic annotation data.

### Scaling to annotate the tree of life

**Figure 1.**
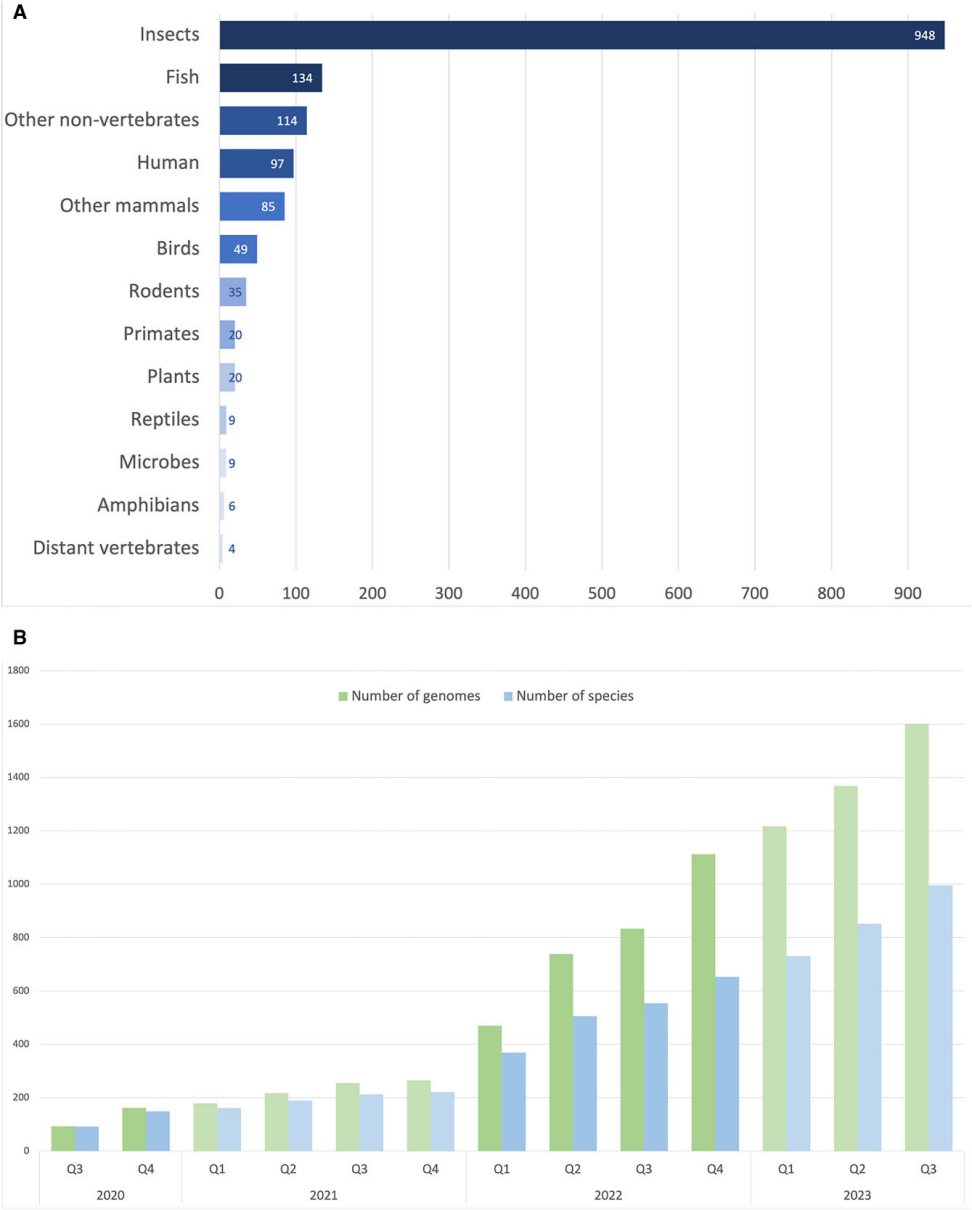
Annotations available on Ensembl Rapid release site. (**A**) Taxonomic breakdown and numbers of all annotations (‘distant vertebrates’ includes sharks and cartilaginous fish). (**B**) Increasing number of annotations available via Rapid Release since its inception in June 2020. The total number of genomes available each month (green bars) and the total number of unique species (blue bars).

In the past year, we have continued our efforts to support global biodiversity research and have doubled the number of annotations available via our Rapid Release site (Figure 1). We have continued to generate annotations for assemblies from global biodiversity initiatives, such as the Darwin Tree of Life project (4, the Vertebrate Genomes Project (6, and the Earth BioGenome Project (7)) (EBP). Importantly, the provision of annotations from hundreds of related genomes from a clade demonstrates these biodiversity projects’ potential for facilitating detailed comparative genomics studies (8). We have increased the number of non-vertebrate annotations supported, primarily in lepidoptera (594 annotations) and diptera (180 annotations), and also now have the first plant annotated via Ensembl pipelines, yellow toadflax (*Lineria vulgaris*; Figure 1).

Our Cactus alignments now cover more eukaryotic clades and species. We have released alignments for 123 species of fish (Actinopterygii), 218 species of Lepidopteran, 37 wheat genomes, 27 rice genomes, 36 coleoptera genomes, 16 crustacea genomes and a 27 genome alignment of pig breeds and out groups. We also continue to provide a set of homologues for all genomes on our Rapid Release site, while a subset of genomes are included in our orthologue calls and gene trees, available through our Ensembl sites.

Ensembl is continuing to scale the underlying infrastructure and software to realise the ambition to support a reference genome annotation for all eukaryotic species under the Earth BioGenome Project umbrella.

### Accelerating food security research

Continuing human population growth and a rapidly destabilising climate pose a significant threat to the security of our global food system. Agriculture and aquaculture need to adapt to these challenges in order to maintain the benefits they provide to human societies, whilst reducing their environmental impact, conserving biodiversity and flexibly adjusting to changing societal expectations. Adoption of genomics-enabled breeding and management, and an improved ability to predict an animal's phenotype using genotype information are key adaptations that are required to meet these challenges. Ensembl has been a valuable resource for food security research for many years (9,10), enabling researchers to quickly and easily access annotated reference genomes for a range of key crop and animal species. By providing access to datasets, analysis tools, and community-focussed platforms for vertebrates, plants and metazoa, Ensembl is helping scientists to identify genes that are involved in important traits that can lead to the development of varieties and breeds that are more productive, and resistant to pests, diseases and climate change.

#### Animal agriculture and aquaculture

**Figure 2.**
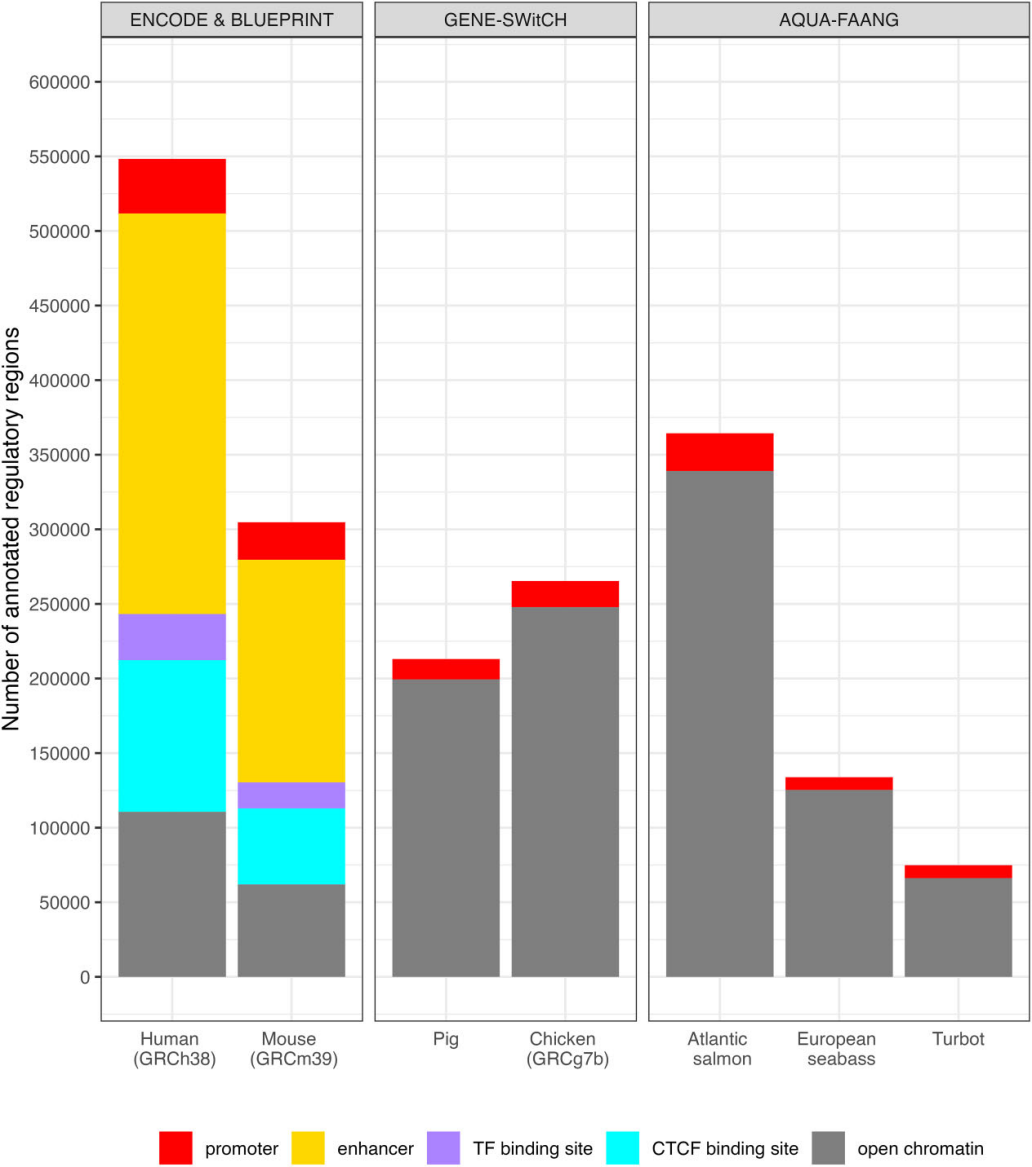
Ensembl provides comprehensive regulatory annotation for human and mouse using data from the ENCODE & BLUEPRINT projects. From Ensembl release 110, regulatory annotation of promoters and open chromatin has been extended to farmed animals. Future work will relabel some of the new open chromatin regions as enhancers based on overlap with relevant histone ChIP-seq peaks.

We have updated key livestock species, annotating the latest assemblies for sheep (*Ovis aries*) and cattle (*Bos taurus*) and have further broadened our coverage by annotating a number of additional pig (*Sus scrofa*) and sheep breeds. This continues our expansion of the number of breeds for each species, important for capturing breed-specific genetic variants to understand more complex traits, particularly associated with diseases, and support genomics-led breeding strategies. This acquisition of multiple references per species leads us towards future pan-genome representations. The latest farmed animal datasets have been made available via the Rapid Release site and will be included on the next main Ensembl site in release, 111. One of the most significant developments for our support of animal agriculture and aquaculture is offering, for the first time, Ensembl regulatory annotation outside of human and mouse in key farmed animal species. Ensembl now offers annotation of promoters and open chromatin regions across different cell types and developmental time points for Pig, Chicken, Atlantic salmon, Turbot and European seabass in collaboration with the GENE-SWitCH (https://www.gene-switch.eu/) and AQUA-FAANG (https://www.aqua-faang.eu/) consortia (Figure 2). Our regulatory build merges open chromatin regions across cell types, which forms the basis for our annotation of candidate regulatory regions. In this initial release, we identify candidate regulatory regions that overlap the start of known transcripts as promoters. In Ensembl release 111 we will relabel some annotated regions as enhancers based on their overlap with relevant histone ChIP-seq peaks. The provision for regulatory region annotation across cell types and developmental time points for key agriculture and aquaculture species is important for researchers to improve genotype to phenotype predictions and to provide further avenues for genomic-led precision breeding to tackle key global food security areas of improved efficiency, disease resistance, adaptation to climate change, and reduced environmental impact.

#### Crop agriculture

We continue to add crop genomes into Ensembl Plants and prioritise the addition of orphan crops alongside major staple crops. Orphan crops are those which typically receive relatively little attention in terms of research, development, and investment compared to major crops, yet often play a crucial role in the diets and livelihoods of communities locally. They provide untapped genetic potential for feeding growing populations in response to climate change, as they often have adaptations to harsh environments and help to diversify diets. We have recently added four additional orphan crops: white fonio (*Digitaria exilis*), cowpea (*Vigna unguiculata*), pigeon pea (*Cajanus cajan*) and teff (*Eragrostis tef*).

Our rice and wheat pan-genome support has increased significantly. We now provide a wheat-specific gene tree and Cactus alignments to all chromosome-level cultivars and wheat relatives. Recently we have added a rice pan-genome (MAGIC 15) as part of the Pan Oryza project. This includes Cactus alignments between the rice varieties and relatives, with a rice-specific gene tree to be released in 111. We have also added genomes for rye (*Secale cereale*), pea (*Pisum sativum*) and two oats (*Avena sativa*) and continued to add further wheat (*Triticum aestivum*) cultivars to Ensembl including recent additions Kariega and Renan.

One of the biggest threats to crop security is destruction through the actions of pests. To provide better resources for analysing crop-pest interactions, we have worked with the Pest Genome Initiative, a collaborative effort between Bayer, Rothamsted Research and Syngenta, supported by the EMBL-EBI Industry programme, to add 21 agriculturally significant insect species to Ensembl. We also expanded the homology annotation in our Rapid Release site, adding three new reference sets so that we now cover Metazoa, Hexapoda, Arthropoda and Protostomia. The co-location of pest and host reference genomes is an important advance for future research in this area and the development of future management strategies.

#### Community support

Ensembl is now a member database of AgBioData (11), a consortium of agricultural biological databases with the mission of consolidating standards and best practices for acquiring, displaying, and reusing genomic, genetic, and breeding data. We are active in steering the direction of activities around data reuse, genome assembly and annotation nomenclature, and standards for genetic variation data.

### Enhancing genomic resources for key species and taxonomic groups

While we have significantly expanded our annotations for species across the tree of life, we continue to provide a rich collection of resources for key species and taxonomic groups.

#### Human and mouse

Human is the most accessed species in Ensembl and a focal point for Ensembl service development and data provision. We continue to refine the human and mouse gene sets with expert manual annotation as part of the GENCODE project. The update of the human genome assembly to GRCh38.p14 allowed us to manually annotate additional ‘patch’ regions, which represent error corrections to the main assembly or novel alternative representations of sequences, especially those that display complex polymorphism. This resulted in the addition of 280 protein-coding genes, containing a total of 2381 transcripts, and 226 pseudogenes within these sequences. The mouse protein-coding gene count has been reduced by 254 to 21 948 as a result of extensive quality control, whose coding capacity had been called into question due to their low scores with PhyloCSF (12), APPRIS (13) and TRIFID (14).

The human X and Y chromosomes have homologous regions, known as the pseudoautosomal regions (PARs). We now separately annotate genes on the Y chromosome, rather than just the X copy, complete with their own Ensembl identifiers. The equivalent genes on chromosomes X and Y are linked in the gene pages on our browser as alleles of the gene on alternate sequences.

The Matched Annotation from NCBI and EMBL-EBI (MANE (15) set of recommended default transcripts for display and variant reporting has been updated to v1.1, which has added 131 new MANE Select and 2 new MANE Plus Clinical transcripts. The percentage of protein coding genes that have a MANE Select has increased from 98.4% in v1.0 to 99.1% in v1.1. These percentages differ to those previously reported as more protein coding genes are now in scope for MANE due to the availability of genes on patches for manual annotation.

To provide a comprehensive summary of the current knowledge for each human variant locus, Ensembl variation and phenotype resources on the GRCh38 assembly are updated in each release. We aggregate data from key sources including dbSNP (16), gnomAD (17), ClinVar (18), DGVa (19), OMIM (20) and the NHGRI-EBI GWAS Catalog (21) and in release 111 will display information for over a billion short variant records and over 7 million structural variants, of which over 2 million and a quarter of a million have phenotype assertions respectively. The resources dbSNP build 156 and ClinVar June 2023 have been updated on GRCh37 to support groups who have not yet made the transition to GRCh38. We have also continued to refine our human regulatory annotation with minor updates such as removing many low confidence CTCF (CCCTC-binding factor) annotations.

#### Vertebrates

We have continued to update the annotation of reference species on the main Ensembl website including updates for Chinese hamster PICR (*Cricetulus griseu*s). We have updated the donkey (*Equus asinus*) reference assembly and annotation and improved the existing horse (*Equus caballus*) annotation using newly generated transcriptomic data. Furthermore we have annotated the latest assemblies for the Norway rat (*Rattus norvegicus*) strains SHR/Utx, WKY/Bbb and SHRSP/BbbUtx as well as the latest assemblies for the naked mole rat (*Heterocephalus glaber*) maternal and paternal haplotigs.

#### Invertebrates, fungi, protists, worms and bacteria

Key reference genome updates include *C. elegans* WormBase (22) annotation WS282, and the latest *D. melanogaster* assembly and annotation, BDGP6.46, both now available in Ensembl. We have also improved our comparative genomics resources, by switching from a single gene tree for all Metazoa, to three covering Metazoa, Protostomia and Insecta. Metazoa and Protostomia gene trees will be updated in every *even numbered* Ensembl release, starting with 110, while Insecta will be released in 111 and then updated in every *odd numbered* release thereafter. We have added 127 new species into Ensembl Metazoa, doubling the number available; two of which were assembled and annotated as part of the Infravec project (23), aimed at developing new vector control measures targeting the greatest threats to human health and animal industries.

We built a new resource to better capture interactions between genes, proteins, mRNA and small molecules across species. Manually-curated and experimentally-verified interactions are imported from PHI-base (24, HPIDB (25) and PlasticDB (26) with exact matches in Ensembl (100% sequence similarity in the exact strain reported in the literature) and are accessible via a new REST API (https://interactions.rest.ensembl.org/) and visualisation on our browser. We have imported 13648 interactions for a total of 470 species across the six Ensembl sites so far. This adds an additional layer of data that enables deeper analysis into the ways species interact in various environments, and the possibility to extrapolate data from known, well-studied species to novel hosts and emerging pathogens.

Four new worm genomes have been introduced into Ensembl Metazoa from our collaborators in WormBase ParaSite. These species are important parasites for humans or livestock, or are used as models for studying parasitism. This includes *Ascaris suum*, a pig roundworm which causes ascariasis and can cross-infect humans.

Ensembl Bacteria has always imported community-submitted annotation from the International Nucleotide Sequence Database Collaboration (27) (INSDC). However, some annotation was inconsistent, out of date and of varying quality. Furthermore, it is now common to submit unannotated bacterial assemblies into INSDC. Our new prokaryotic annotation pipeline, built jointly with MGnify (28), provides a consistent and scalable method for both isolates and Metagenome-Assembled Genomes (29) (MAGs). We have re-annotated all our bacteria (except for 115 species which have retained the previous community-submitted annotation) and developed a systematic naming strategy for these new genes based on a digest of key gene attributes including its genomic position, the species and underlying contig; an approach similar to the scheme employed by the Variation Representation Specification (30).

### Ensembl genome interpretation and tool improvements

#### Ensembl variant effect predictor (VEP)

Increasing numbers of human genomes are being sequenced in clinical diagnostics and research laboratories but interpretation of novel variants remains challenging. To help address this, we have further extended Ensembl VEP’s (5) functionality for the annotation and prioritisation of genomic variants. Efforts are underway to use highly parallel assays to investigate the effect on cellular phenotypes of all possible variants in specific functional regions. This work has the potential to revolutionise basic research, patient diagnosis and drug discovery, but standards for data description and exchange as well as simple access methods are needed. Groups adopting these approaches are collaborating to create an Atlas of Variant Effect (31) and submit their results to MaveDB (32). We have integrated results from MaveDB into Ensembl VEP, where they can be easily accessed via the user-friendly web interface, REST API and command line package. Tools utilising advanced Artificial Intelligence approaches are now improving the prediction of variant deleteriousness. We have developed an Ensembl VEP extension to integrate pre-calculated scores from DeepMind's Enformer algorithm (33), which predicts when variants are likely to impact gene regulation. To highlight when variants have been previously reported in people with Mendelian disease, we have also integrated Geno2MP (34) into Ensembl VEP and provide links from the web interface to enable further exploration.

We have further enhanced Ensembl VEP options for the annotation of structural variants (SVs), which continue to be investigated in population and disease–association studies. We have increased the range of SVs for which molecular consequence is predicted to include breakends and to take copy number into account for copy number variants. We have also updated Ensembl VEP’s algorithm to identify overlapping reference features to enable more rapid, configurable matching to reference SV datasets and enable annotation with population frequency data (e.g. gnomAD) or assertions of clinical significance (ClinVar). While there is a profusion of tools designed to predict the likely deleteriousness of single nucleotide variants, there has been less work on the evaluation of SVs. One method in this area is CADD-SV (35), which scores the likely deleteriousness of large human duplications insertions and deletions using knowledge of functional regions in the genome and chimpanzee data in a random forest model. We have enabled the integration of precalculated CADD-SV scores into Ensembl VEP for ease of use.

We are engaged with relevant communities, working with the European Joint Programme on Rare Diseases to accelerate rare disease investigations by identifying and delivering new variant analysis functionality and improving tool integration, and with the GA4GH on the development of improved standard for data exchange. We have implemented the GA4GH Beacon v2 (36) specification to enable the retrieval of rich variant annotations and enable the improved interoperability of our data.

#### The new Ensembl website

The Ensembl Beta site (https://beta.ensembl.org/) and associated infrastructure continues to provide the community with access to the new interfaces and feature developments. We continue our informed and iterative approach to its design by engaging with existing users through interviews and surveys.

**Figure 3.**
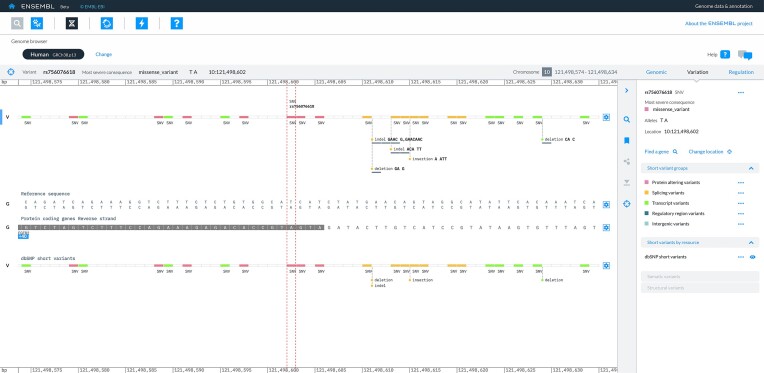
Novel rendering of variants in new Ensembl website. rs756076618, a short variant from dbSNP that lies close to a splice site in the MANE Select transcript for FGFR2, has been selected as the focus object. Variants are coloured according to group, and the browser can optionally be configured to display identifiers, alleles and the extent of the reference sequence affected.

New tracks added to the genome browser this year include regulatory annotation for human GRCh38 and variant tracks for *Triticum aestivum*, *Saccharomyces cerevisiae*, GRCh38 and GRCh37. Individual variants can now be set as the focus of the genome browser (Figure 3), allowing fast access to more detailed information, which is retrieved from a dedicated in-house API. Other developments include a redesigned gene search and BLAST tool, with the latter providing a distinct presentation of BLAST search hits against transcripts compared to genomes.

#### Tark and GraphQL

We have added new Ensembl releases to The Ensembl Transcript Archive (Tark; https://tark.ensembl.org/) and it now has data available in it up to and including Ensembl release 110 (https://tark.ensembl.org/web/datatable/release_set/). As part of the ongoing work to redevelop and refresh the Ensembl infrastructure, a GraphQL service (https://beta.ensembl.org/data/graphql) has been created to provide programmatic access to Ensembl data. The service provides data for genes, transcripts, proteins, associated metadata and genomic locations for all genomes available through our beta infrastructure.

### Outreach and training

Ensembl offers a varied training programme including in-person and synchronous virtual courses as well as asynchronous online courses (https://www.ebi.ac.uk/training/on-demand). We continue to deliver virtual courses with open registration for participants across the globe while also collaborating with host organisations to provide training for specific communities, tailored to their needs and interests. Course materials are distributed through our training site (https://training.ensembl.org/). We continue to offer personalised assistance with specific Ensembl queries on our helpdesk, helpdesk@ensembl.org, and through our developer mailing list (https://lists.ensembl.org/mailman/listinfo/dev).

### Future directions

Over the next year we will continue to scale to support an increasing breadth of eukaryotic reference annotations under the Earth BioGenome Project umbrella. We will also continue to deepen the number of reference annotations available per species as we advance towards pan-genome representations of key species, driven by our involvement in projects such as the human pangenome reference consortium and agricultural initiatives in crops and farmed animals. The increasing presentation complexity of the breadth and depth of our genomic annotations continues to drive the features of the future Ensembl platform. The new Ensembl website, currently in beta version, will soon release data for nearly 250 assemblies together with new interfaces for finding and managing species of interest and for discovering homology relationships between genes. We will continue to increase the number of species, aiming to replace our Rapid Release site during 2024. We are communicating closely with agricultural consortia that are organising new telomere to telomere reference assemblies, following advances in human, so that Ensembl can rapidly annotate these additional reference assemblies as needed. A key expected development, particularly for biodiversity, is the European Nucleotide Archive (37) supporting submission of decoupled annotation files in GFF3 format. This will be an important milestone for disseminating our own annotations globally through the public INSDC archives, but also an important step towards better support for the representation of community produced annotations in Ensembl. We will continue to expand annotations across the tree of life, providing openly accessible, comprehensive genomic information through an easy-to-use interface complete with a range of powerful tools and services.

## Data Availability

All Ensembl integrated data are available without restriction from the main website (https://www.ensembl.org), the Rapid Release site (https://rapid.ensembl.org), in bulk from the FTP site (https://ftp.ensembl.org) and programmatically via the REST API (https://rest.ensembl.org). A documented overview of our different genome annotation methodologies is available at https://rapid.ensembl.org/info/genome/genebuild/index.html, with the type of annotation produced indicated on each species’ home page. Ensembl code is available from GitHub (https://github.com/Ensembl) under an open source Apache 2.0 licence. News about our releases and services can be found on our blog (https://www.ensembl.info), our announce mailing list (https://lists.ensembl.org/mailman/listinfo/announce), Twitter (@ensembl; https://twitter.com/ensembl) and Facebook (https://facebook.com/Ensembl.org).
